# Pneumococcal prophages are diverse, but not without structure or history

**DOI:** 10.1038/srep42976

**Published:** 2017-02-20

**Authors:** Angela B. Brueggemann, Caroline L. Harrold, Reza Rezaei Javan, Andries J. van Tonder, Angus J. McDonnell, Ben A. Edwards

**Affiliations:** 1 Nuffield Department of Medicine, University of Oxford, United Kingdom

## Abstract

Bacteriophages (phages) infect many bacterial species, but little is known about the diversity of phages among the pneumococcus, a leading global pathogen. The objectives of this study were to determine the prevalence, diversity and molecular epidemiology of prophages (phage DNA integrated within the bacterial genome) among pneumococci isolated over the past 90 years. Nearly 500 pneumococcal genomes were investigated and RNA sequencing was used to explore prophage gene expression. We revealed that every pneumococcal genome contained prophage DNA. 286 full-length/putatively full-length pneumococcal prophages were identified, of which 163 have not previously been reported. Full-length prophages clustered into four major groups and every group dated from the 1930–40 s onward. There was limited evidence for genes shared between prophage clusters. Prophages typically integrated in one of five different sites within the pneumococcal genome. 72% of prophages possessed the virulence genes *pblA* and/or *pblB*. Individual prophages and the host pneumococcal genetic lineage were strongly associated and some prophages persisted for many decades. RNA sequencing provided clear evidence of prophage gene expression. Overall, pneumococcal prophages were highly prevalent, demonstrated a structured population, possessed genes associated with virulence, and were expressed under experimental conditions. Pneumococcal prophages are likely to play a more important role in pneumococcal biology and evolution than previously recognised.

Infectious diseases are a leading cause of early childhood deaths, among which pneumonia is the most common: an estimated 1.3 million children died of pneumonia in 2013. The leading cause of pneumonia was *Streptococcus pneumoniae* (the pneumococcus), which is also a major cause of paediatric meningitis and bacteraemia. Children who survive life-threatening pneumococcal diseases often have profound disabilities^1^,^2^.

Safe and effective pneumococcal vaccines are available to prevent disease, but vaccines are not yet universally administered among all countries, particularly those in the geographical regions in need of the greatest protection. Furthermore, although they are safe and effective, current vaccines are limited in the protection they provide. Antimicrobials can be used to treat pneumococcal infection, but antimicrobial-resistant pneumococci are found worldwide and resistance can result in treatment failures. Therefore, despite the availability of vaccines and antibiotics, pneumococcal disease remains a major global challenge^3^,^4^,^5^. Crucially, a long-standing unresolved question is what determines pathogenicity, because pneumococci predominantly reside in the healthy paediatric nasopharynx and it is still unclear why some pneumococci cause devastating disease and others do not.

Phages infect bacteria by attaching to surface-exposed host receptors and injecting their DNA into the cell. There are virulent phages that are only lytic, and temperate phages that can be both lytic and lysogenic. In the lytic cycle, phages synthesise new progeny, cause the host to lyse and release new viruses, while in the lysogenic stage, the viral genome is stably integrated into the bacterial DNA and replicates along with the bacterial chromosome. Prophages (temperate phage DNA that is integrated within the bacterial genome) can become lytic in a process called induction, which is triggered spontaneously or by external stimuli such as exposure to mitomycin C. Prophages are one type of mobile genetic element, transferring DNA to and from the bacterial genome^6^,^7^.

Expressed prophage genes often have a phenotypic effect on the host bacterium, for example by producing a toxin that increases bacterial virulence (*Vibrio cholerae* and *Staphylococcus aureus*), enhancing bacterial adherence to platelets (*Streptococcus mitis*), or evading immune defences (*Pseudomonas aeruginosa*)^6^,^7^,^8^. Importantly, such genes may be functional even if the prophage is defective or the prophage genome is incomplete. Host bacteria can also be infected with multiple prophages: for example, one strain of *Streptococcus pyogenes* contains six different prophages or prophage-like elements that together comprise approximately 12% of the bacterial genome and have been shown to directly contribute to the high virulence of that lineage^9^.

Pneumococcal phages were first reported in 1975 and subsequent work using simple DNA probe-based detection of one prophage-associated gene suggested that the majority of pneumococcal genomes contain prophages; however, prevalence data has been limited and little is known about how a prophage affects the pneumococcal host^10^,^11^,^12^,^13^. We showed that a globally-distributed multidrug-resistant pneumococcal lineage called PMEN1 likely gained some of its epidemiological success from the acquisition of three mobile genetic elements including the MM1 prophage, which has been shown *in vitro* to enhance adherence to human cells^14^,^15^,^16^. We also discovered a surprising link between prophages and antibiotic resistance: a pneumococcal prophage harboured the genes that confer tetracycline resistance^17^.

Prophages are widespread and demonstrably linked to increased infection, pathogenesis and virulence in many bacterial species. Prophages are likely to be highly prevalent within the pneumococcal population, but the genetics, diversity and molecular epidemiology of pneumococcal prophages are not well understood. If pneumococcal prophages are widely distributed, genetically highly diverse and frequently recombining then it will be a challenge to associate specific prophages with pneumococcal genotypes and phenotypes. Alternatively, if pneumococcal prophages are highly prevalent but the prophage population is structured, then there will be a framework on which to investigate specific prophage/pneumococcus interactions and prophage-derived mechanisms that may contribute to pneumococcal phenotype, disease potential and epidemiological success. Uniquely, we have included a set of historical genomes dating from 1916 onwards, allowing us to investigate the potential for the persistence of prophages over many decades.

Therefore, the aims of this study were to: i) determine the prevalence of prophage DNA within a large global and historical pneumococcal genome dataset; ii) assess the genetic diversity and molecular epidemiology of prophages among pneumococci isolated over a 90 year period; and iii) investigate associations between these prophage sequences and pneumococcal genetic lineages.

## Results

### High prevalence of prophage sequences among a diverse collection of pneumococcal genomes

The genomes analysed in this study comprised a diverse set of 482 pneumococci recovered between 1916 and 2008 from people of all ages residing in 36 different countries. Pneumococci were recovered from both healthy individuals and those with disease. 91 pneumococcal serotypes, 258 multilocus sequence types (STs) and 94 different clonal complexes (CCs; clusters of closely-related pneumococci) were represented in the dataset (Table S1).

**Figure 1:**
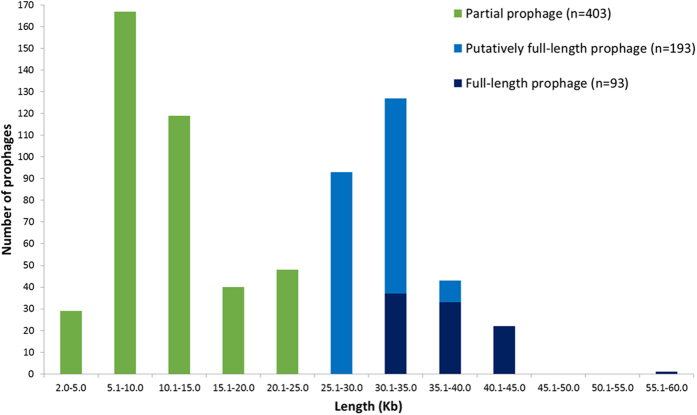
Graph depicting the number of prophages in each category: partial, putatively full-length or full-length. Bars represent the number of prophages relative to the length of the prophage sequence that was identified.

Overall, 100% of the pneumococcal genomes contained coding sequences annotated as being phage-associated. In particular, 689 full-length, putatively full-length and partial prophage sequences at least 2 Kb in length (see Methods for definitions) were identified among 326 (68%) of the 482 pneumococcal genomes (Fig. 1). Phage sequences <2 Kb in length were not investigated further in this study. The total amount of prophage sequence (≥2 Kb in length) within a single pneumococcal genome ranged from 4–135 Kb, which was up to 6% of the pneumococcal genome.

In total, we revealed 163 previously unreported full-length or putatively full-length pneumococcal prophages in this dataset. 93 (66 representative examples) full-length prophage sequences were identified and these were 29–60 Kb (median 37.5 Kb) in length (Fig. 1; Table S2). 76 (56 representative examples) of these full-length prophage sequences were new. 193 putatively full-length prophages ranged in size from 25–38 Kb; 129 of these sequences were unique and 107 of the unique prophage sequences were new (12 matched to prophage sequences found in GenBank and 10 matched to new full-length prophages in this study). In addition, 403 partial prophage sequences were identified among the 482 pneumococcal genomes and these ranged in length from 2–25 Kb.

45% (219/482) of the pneumococcal genomes harboured at least one full-length and/or putatively full-length prophage sequence. Among these 219 genomes, 28% (n = 63) were poly-lysogenic, i.e. contained >1 full-length or putatively full-length prophage sequence within the pneumococcal genome.

### Prophage sequence alignments demonstrate clusters of prophages that persist for decades

**Figure 2:**
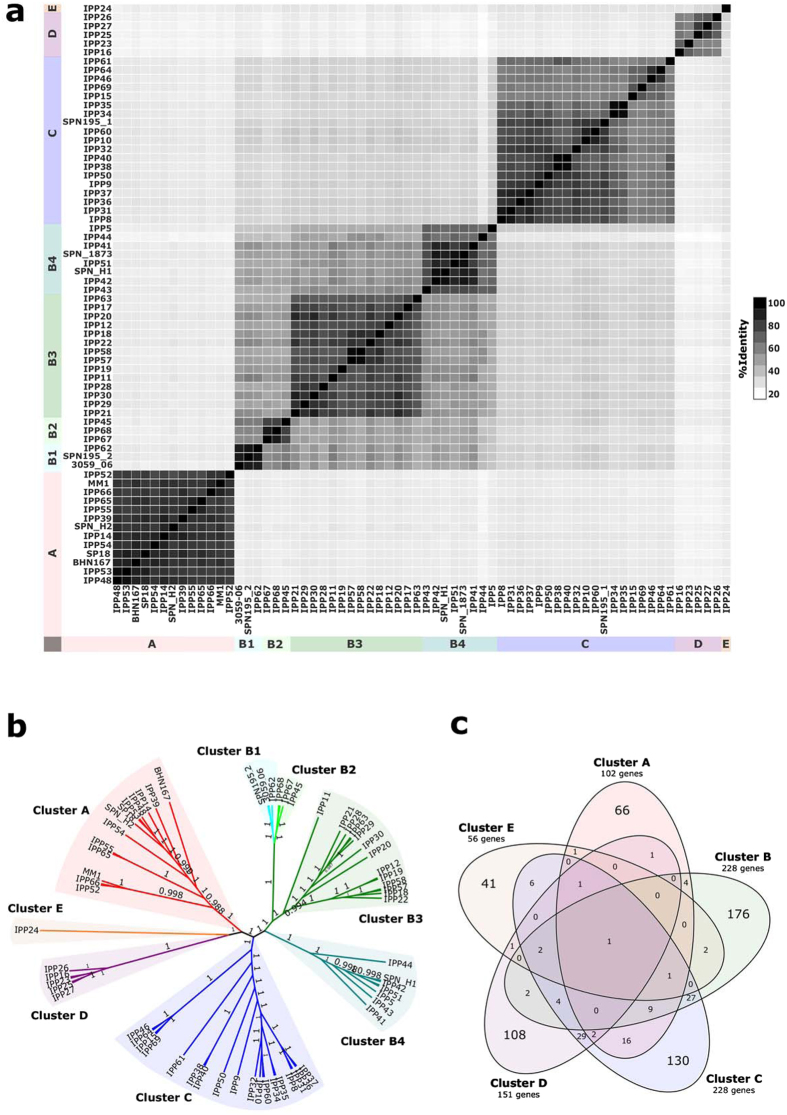
Description of nucleotide sequence similarity, phylogenetic clustering and shared genes among the 66 representative full-length prophages identified in the pneumococcal genome dataset. (**a**) Heat map depicting the percentage nucleotide sequence identity shared between pairs of full-length prophage sequences. Groups of similar prophages are marked A-E and correspond to the clusters seen in part b. (**b**) Clusters of prophage sequences based upon nucleotide sequence identity. Bootstrap values are marked on branches of each cluster. (**c**) Venn diagram depicting genes unique to each prophage cluster and genes shared between clusters.

The 66 representative full-length prophage sequences were aligned and the average pairwise identity was only 40.2% (range 20.8–97.4%); however, pairwise comparisons of prophage sequences and an unrooted phylogenetic tree depicted four major prophage clusters and one single prophage (Fig. 2a,b and Figure S1). Remarkably, all four major clusters included prophages integrated in host pneumococci that were recovered in the 1930–40 s as well as among modern pneumococci, demonstrating the persistence of the prophage clusters and some prophages over several decades.

The evidence for genes shared between prophage clusters was limited. Figure 2c depicts all of the different genes (<90% amino acid sequence similarity) identified among all representative prophages of each cluster, and whether any of those genes were found among any prophage(s) of any other cluster. Small numbers of individual genes were shared between some prophages in different clusters, e.g. one holin gene sequence was found in 19 prophages distributed across all five clusters (Fig. 2c and Table S3); however, the vast majority of genes within a prophage cluster were only found among that cluster of prophages (Fig. 2c). There were three larger groups of shared genes (cluster C and either cluster A (n = 16), B (n = 27) or D (n = 29), respectively) and generally these were sets of genes, sometimes contiguous genes, which were shared by a subset of prophages from two clusters (Table S3). The majority of the shared genes were predicted to encode proteins with unknown functions.

**Figure 3:**
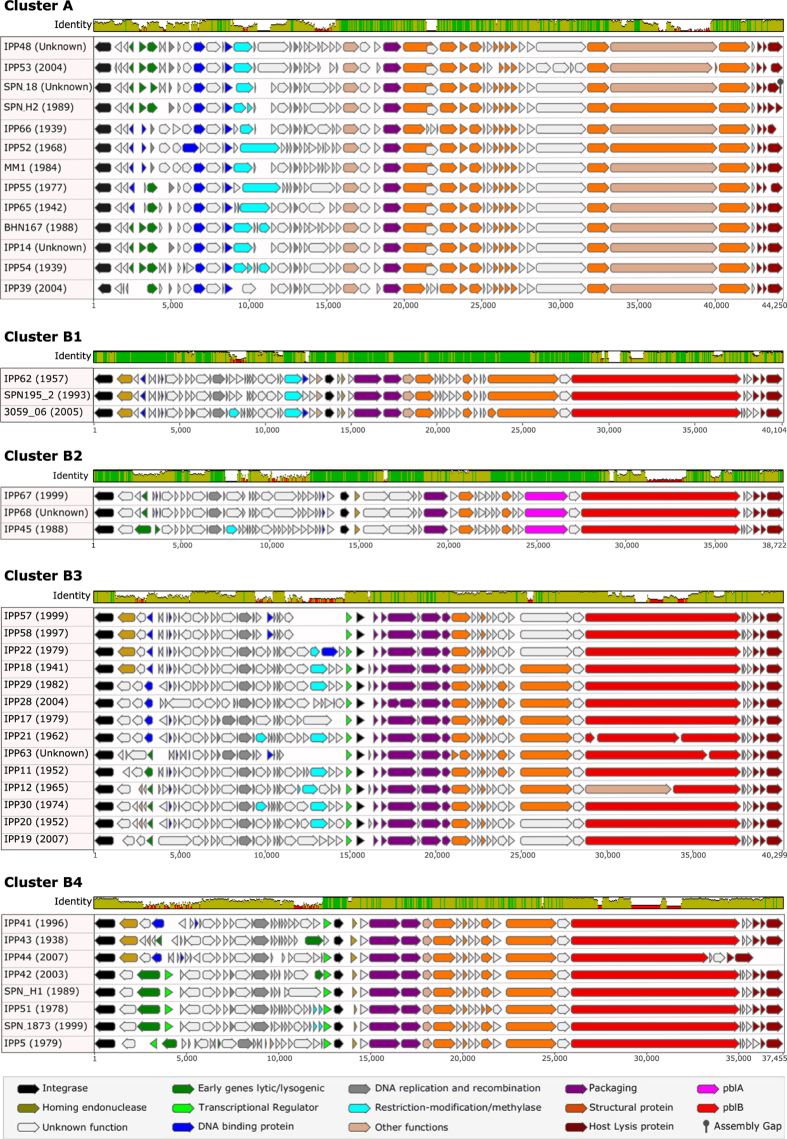
Nucleotide sequence alignments of representative full-length prophages from clusters A and B. The coloured bar at the top of each cluster indicates the mean pairwise nucleotide sequence identity over all pairs in the column: bright green = 100% identity; green-brown = <100% but >30% identity; and red = <30% identity. Prophage genes are coloured based on putative or known function. The names of each prophage are given, followed by the year of isolation (in brackets) of the oldest known pneumococcus harbouring that prophage.

**Figure 4:**
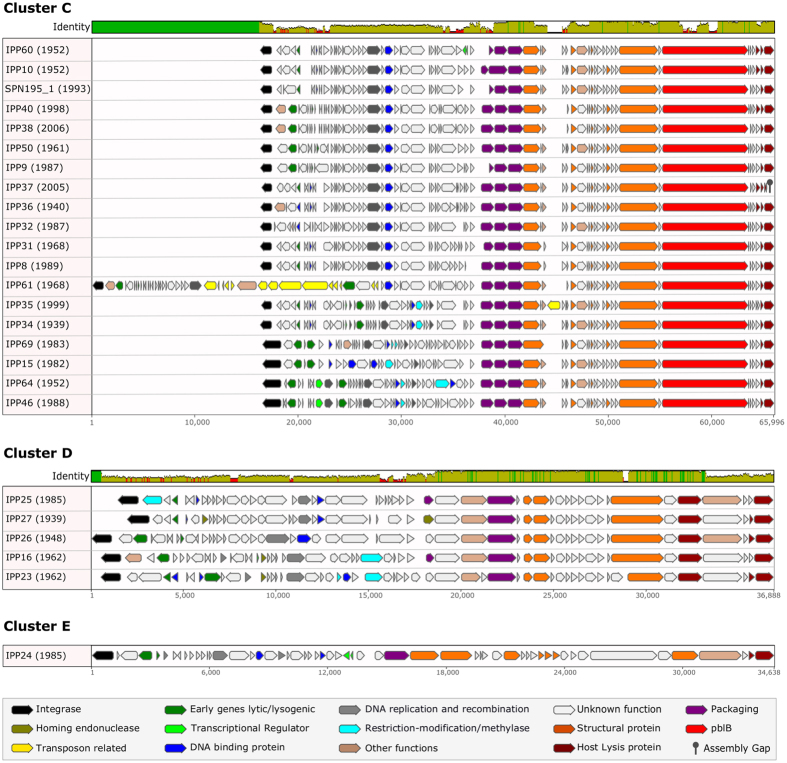
Nucleotide sequence alignments of representative full-length prophages from clusters C, D and E. The coloured bar at the top of each cluster indicates the mean pairwise nucleotide sequence identity over all pairs in the column: bright green = 100% identity; green-brown = <100% but >30% identity; and red = <30% identity. Prophage genes are coloured based on putative or known function. The names of each prophage are given, followed by the year of isolation (in brackets) of the oldest known pneumococcus harbouring that prophage.

Prophages within every cluster possessed genes involved in DNA packaging, the production of the phage capsid, tail and other structural proteins, and genes encoding hypothetical proteins that were identical or highly similar (>95% identical at a nucleotide level) to the other prophages in that cluster (Figs 3 and 4). Prophage sequences also possessed a cluster-specific set of other identical and highly similar genes including those that encoded the integrase, other enzymes, DNA binding and replication proteins, holin proteins and/or lytic amidase (Figs 3 and 4; Table S4).

Thirteen representative cluster A prophages had an average pairwise nucleotide sequence identity of 83.6% (range 75.9–95.1%; Fig. 3). These were identified among 15 pneumococci recovered from 1939–2005 (Table S2). Cluster B prophages (n = 28) subdivided into 4 smaller clusters (Figs 2a,b and 3). Clusters B1 and B2 each depicted three representative prophages: cluster B1 prophages shared 91.9% average pairwise identity (range 90.6–93.8%) and were found in three different pneumococci recovered in 1957, 1993 and 2005; cluster B2 prophages were 78.9–91.8% identical (average 83.3%) and were identified in five host pneumococci from 1988–2000 (Fig. 3 and Table S2). Fourteen representative cluster B3 prophages (average pairwise identity of 77.9%, range 70.2–96.8%) were identified among 21 pneumococci recovered from 1941–2007. Eight representative cluster B4 prophages averaged 78.7% pairwise identity (range 66.1–95.3%) and were found in 13 pneumococci recovered from 1938–2007 (Table S2).

Nineteen representative cluster C prophages were identified among 21 pneumococci recovered from 1940–2008 and shared only 69.6% (range 55.1–97.4%) pairwise identity overall (Fig. 4 and Table S2). Cluster C included the IPP61 prophage sequence that harboured the tetracycline resistance mobile genetic element^17^,^18^, which was not present in any of the other prophages. Five representative cluster D prophages and one cluster E prophage were also more diverse: the overall pairwise identity among cluster D prophages was 65.6% (range 57.8–81.9%) and the five host pneumococci were identified from 1939–1985. The cluster E prophage, IPP24, was identified in a host pneumococcus from 1985 (Fig. 4).

### Relationships between prophage integration sites and prophage integrase sequences

The 93 full-length prophages were consistently integrated in specific locations of the pneumococcal genome. There were five major categories of integration sites, within which the patterns of pneumococcal genes flanking the prophage integrase and amidase genes were consistent (Table 1). The flanking pneumococcal genes were involved in transcriptional regulation, metabolic enzyme production and/or activity, purine nucleotide biosynthesis, regulation of DNA repair, and competence. The 193 putatively full-length prophages were inserted in the same five major insertion site categories (Table S5).

The integration site was directly associated with the nucleotide sequence of the prophage integrase and the individual prophage clusters (Table 1). Based upon a threshold of ≥90% nucleotide sequence identity, the 93 full-length prophage integrase sequences clustered into two major groups (I and II) that together described 75.5% of the prophage integrases. There were also two minor integrase groups (III and IV), identical integrase sequences in a pair of prophages, and four single prophages that each had a unique integrase sequence. There was little to no sequence similarity between integrase groups.

Cluster A prophages were split in terms of integrase groups: seven prophages (IPP48, IPP53, SPN_18, SPN_H2, IPP66 and IPP52) had a group I integrase and six (MM1, IPP55, IPP65, BHN167, IPP14, IPP54 and IPP39) possessed a group III integrase. All cluster B prophages possessed an integrase from group II, apart from IPP5, which had a unique integrase. Cluster C prophages mainly had an integrase from group I, apart from four prophages (IPP64, IPP46, IPP15 and IPP69) that had a group IV integrase. One prophage in cluster D (IPP26) had a group I integrase sequence whereas the four others possessed unique integrase sequences. The single prophage IPP24 contained an integrase sequence from group I.

### *pblA* and *pblB*, putative virulence factors

The phage tail is used for host recognition, attachment and delivery of the phage DNA to the host cell. *pblA* and *pblB* are phage tail protein genes that were first described in *S. mitis* and were shown to be associated with platelet binding and an increased risk of endocarditis^8^. Pneumococcal prophage-associated *pblB* was recently shown to be associated with pneumococcal adherence to human lung epithelial cells and increased persistence in the nasopharynx and lung in a murine model of pneumococcal infection^19^.

In the current study, only prophages in clusters B and C (71.6% of the 66 unique full-length prophage sequences) possessed *pblA* and/or *pblB* (Figs 3 and 4). The three prophages in cluster B2 were the only prophages that possessed *pblA* and those *pblA* sequences were virtually identical (99.9% pairwise identity), although the pairwise sequence identity among the three versions of *pblB* possessed by these three prophages was only 67.1%. Overall, *pblB* sequence diversity was high, which was perhaps not surprising since the phage tail interacts with the host. The average pairwise sequence identity was 63.5% among all 47 *pblB* sequences, although within-cluster sequence identity was higher: clusters B1 (83.6%); B2 (67.3%); B3 (68.0%); B4 (69.6%); and C (79.7%). The sequence variation within *pblB* led to sequencing challenges associated with this particular gene, i.e. the majority of the putatively full-length prophage sequences in this study had a fragmented *pblB* and as a consequence, an incomplete sequence assembly. There were no complete matches to the *pblB* sequence from the murine model study (NTUH-P15 *pblB*)^19^: the best matches to any prophage *pblB* in this study were 97–98% similar matches to only ~1.2–4.4 Kb of the NTUH-P15 *pblB* sequence (data not shown).

### Molecular epidemiology and persistence of full-length and putatively full-length prophage sequences

**Figure 5:**
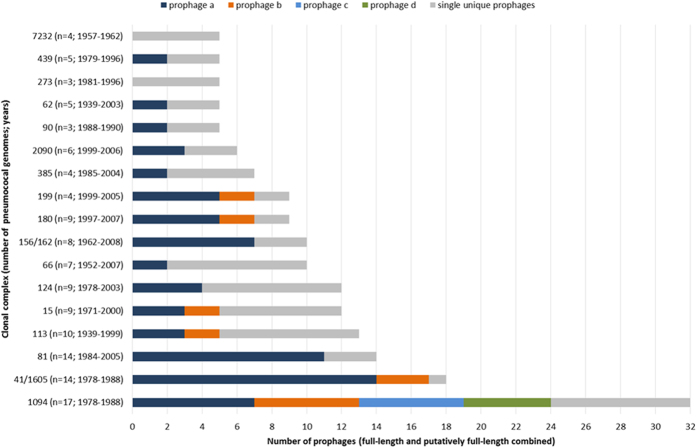
Illustration of the number of different full-length and putatively full-length prophages identified in each of the major pneumococcal clonal complexes. Clonal complexes are labelled on the y-axis followed by brackets containing the number of pneumococcal genomes within that complex and the years of isolation of those pneumococci. See Table 2 and S2 for details of the specific prophages identified within each clonal complex.

Collectively, the 286 full-length and putatively full-length prophages were identified among 56 different CCs and 36 Singletons (unclustered pneumococci) overall. Five or more prophages were identified among the pneumococci representing 17 different CCs (Fig. 5). The two CCs with the greatest number of prophages, CC1094^6A^ (CC^serotype^; n = 32) and CC41/1605^19A^ (n = 18), were lineages of pneumococci recovered in South Africa during the 1970–80 s, apart from one pneumococcus in CC41/1605^19A^ that was recovered in the USA in 1985. Twelve different prophages were detected among the 17 pneumococci in CC1094^6A^ and 15 of the 17 pneumococci each contained two different prophages. Three different prophages were detected among 14 pneumococci in CC41/1605^19A^ and four of the pneumococci each contained the same pair of prophages. The pneumococci of CC1094^6A^ and CC41/1605^19A^ were isolated from both healthy people and those with invasive disease. CC1094^6A^ remains a major pneumococcal lineage causing invasive disease in South Africa^19^, but whether contemporary pneumococci of CC1094^6A^ continue to maintain these prophages is currently unknown.

CCs 113^18C^, 15^14^, 180^3^ and 199^19A^ are all globally-distributed lineages of pneumococci (reference strains PMEN36, PMEN9, PMEN31, and PMEN37, respectively) that are prevalent in carriage and disease worldwide^20^,^21^. Each of these lineages possessed two predominant prophages plus other single prophages (Fig. 5). In contrast, CCs 81^23F^ and 156/162^9V^ are also globally-distributed lineages (PMEN1 and PMEN3, respectively) in disease and carriage, but the majority of pneumococci in these CCs harboured one main prophage.

Furthermore, there was generally a strong association between individual prophages and the host pneumococcal genetic lineage, that is, unique prophages were usually found in one (or one predominant) CC. Nineteen different prophages were found at least three times within the entire dataset and of these, 13 prophages were found exclusively in a single CC (Table 2). Most notably, some of these prophages persisted for decades: IPP34 (CC113^18C^; ≥60 y); IPP12 (CC615^1^ and CC217^1^, ≥54 y); IPP29 (CC124^14^; ≥24 y); and MM1 (CC81^23F^; ≥18 y). Serotypes 18C, 1 and 14 have a high invasive disease potential^22^, but whether the specific prophages associated with these serotypes and genetic lineages contribute to pathogenesis remains to be determined. There was no obvious association between specific prophages and pneumococcal serotypes, independent of the well-recognised serotype association with CC (Table S2)^20^.

### RNA sequencing demonstrated clear evidence for prophage gene expression

**Figure 6:**
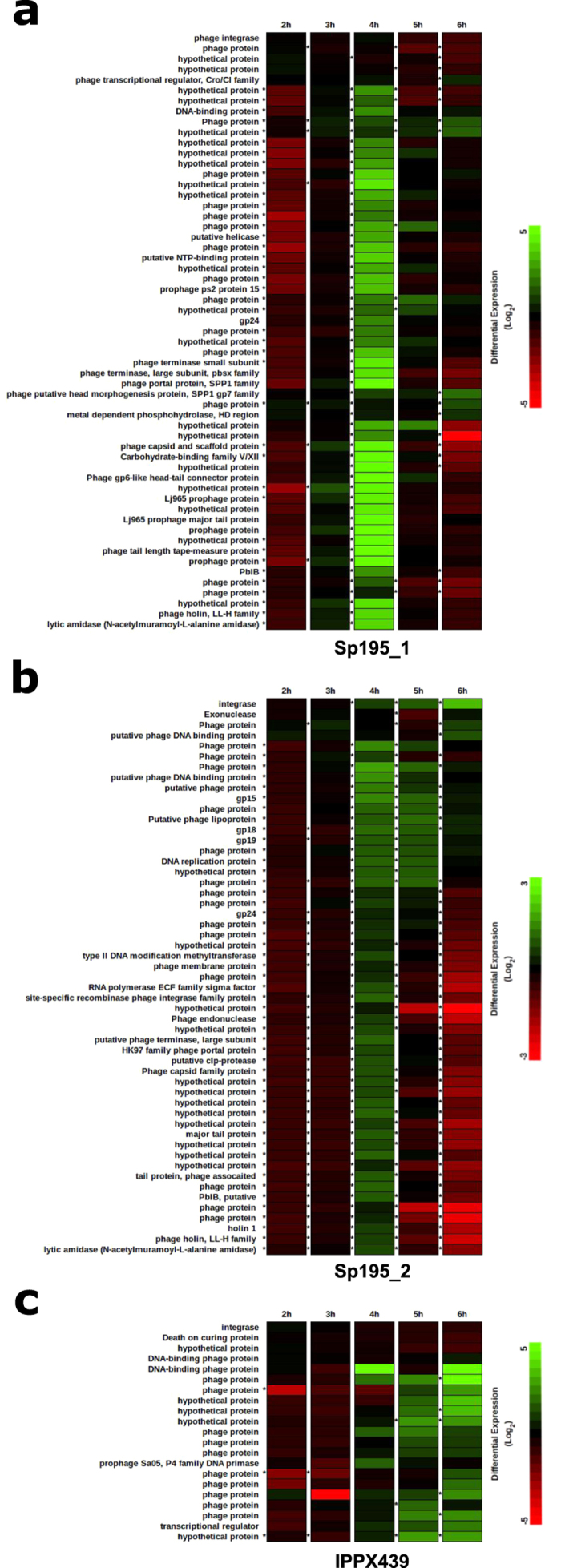
Heat maps describing the results of the RNA-seq experiment. Prophage genes are depicted by rows and differential expression levels at each of five time points are presented in columns. An asterisk to the left of a cell indicates a statistically significant differential level of expression (p < 0.05). Prophage gene expression levels are given for two full-length prophages, Sp195_1 (**a**) and Sp195_2 (**b**), and one partial prophage sequence, IPPX439 (**c**). Mitomycin C was added to the broth culture after 3 h of incubation.

RNA sequencing was utilised to explore whether prophage gene expression could be detected. The pneumococcal reference strain PMEN3 (Spain^9V^−3), which contained two full-length prophages and one partial prophage, was grown in broth culture and mitomycin C was added to prompt prophage induction. PMEN3 culture samples were taken at sequential time points and RNA was extracted and sequenced. The RNA-seq data clearly showed prophage gene expression among all three prophage sequences (Fig. 6). Both full-length prophages significantly expressed nearly their full complement of genes after mitomycin C induction, while downregulating the genes involved in integration and lysogeny. Remarkably, most of the genes associated with the partial prophage sequence also were expressed after the addition of mitomycin C, and some of the gene expression levels were also statistically significant. The functions of nearly all of the partial prophage genes are currently unknown.

## Discussion

The high prevalence of prophages among pneumococci was predicted some years ago, but the advent of affordable genome sequencing has made it possible to more easily identify prophages. That said, a rapid and straightforward identification of the many prophages in this study by screening tools and pipelines was hindered since so many of the prophages were newly discovered. This study required significant manual effort and inspection of sequences, but the return was a lengthy list of prophages to investigate here and in future studies. The key overall finding was that the breadth and depth of prophage sequences within pneumococcal genomes was astonishing.

The second most important finding was that the pneumococcal prophage population does appear to be structured. Many papers describe the vast diversity of phages among bacterial species – and high phage diversity is unequivocal^23^ – but our data and analyses also clearly demonstrated clusters of closely-related pneumococcal prophages and in some cases, clear associations of specific prophages with pneumococcal genetic lineages. Population structure (genetic clustering) in bacterial populations reveals clusters of closely-related individuals and suggests that the population is diversifying from a common ancestor, and this diversification might lead to a beneficial outcome like increased virulence or antibiotic resistance. The pneumococcal population structure is reasonably well understood in terms of major circulating lineages and their association with disease, carriage, antibiotic resistance, geographical location, and so on. The fact that prophages can be associated with specific genetic lineages within the pneumococcal population provides a framework on which to assess the specific contribution prophages might make to a genetic lineage, particularly in the context of increased cell adherence, pathogenesis, virulence, etc. Additionally, the function of many of the prophage genes is unknown and thus these prophages represent a vast reservoir of new information to be revealed.

Furthermore, although the prophage genes were organised in ‘modular’ groups, e.g. genes encoding phage structural components were located adjacent to each other in the prophage sequence, we could find relatively little evidence (given the large numbers of prophages and prophage genes investigated in this study) for genes that were similar at a sequence level and shared between prophage clusters. This is relevant because one theory of phage evolution is that modular groups of genes are exchanged between different phages; however, that does not appear to be an accurate reflection of evolution among pneumococcal prophages^24^. More broadly, this study highlighted the importance of analysing a large collection of diverse bacterial genomes to reveal the similarities and differences among prophages within the same bacterial species.

The third major finding was that some prophages persisted over long time periods – sometimes relatively unchanged at a nucleotide sequence level. Given the high level of recombination and frequent exchange of genetic material between unrelated pneumococci, this suggests that some form of selective pressure may be acting to maintain the integration of certain prophages within specific pneumococcal lineages. Moreover, the prophages represent large regions of DNA and therefore the maintenance of so much foreign DNA could suggest that the prophages provide a benefit to the host pneumococcus.

Furthermore, RNA sequencing of the prophages found in the reference strain of one globally-distributed, multidrug-resistant pneumococcal lineage clearly demonstrated prophage gene expression in a simple *in vitro* experiment. At a minimum, this provided evidence for a functional set of prophage genes and proof-in-principle that RNA sequencing could be a useful experimental tool to understand prophage gene expression. It remains to be shown whether functional phage proteins are produced *in vivo*, but the potential for RNA sequencing to contribute to our understanding of pneumococcal prophages is evident.

It is also important to note that the full-length prophage prevalence in our study was almost certainly underestimated. Some of the older genomes sequenced by us and others were comprised of many assembly contigs; this made full-length prophage sequence identification very challenging and for this reason we resequenced some of our older genomes to improve sequence quality. We could not do this for all pneumococci, thus will have missed some full-length prophage sequences if they were split over multiple contigs and the presence of other prophage sequences in the same genome meant that correct prophage assemblies could not be guaranteed. DNA sequencing technology and genome assembly tools are continually improving and newer genome assemblies are comprised of only a few contigs, meaning that incomplete prophage sequences will be less frequent and problematic going forward.

The pneumococcal genome collection was compiled to capture population-level diversity and uniquely investigate historical pneumococci, but much of the pneumococcal metadata were unknown and thus it was difficult to associate specific prophage(s) and pneumococcal phenotype from this dataset. However, our work provides the foundation for future studies to explore associations between unique prophages and pneumococcal phenotypes among well-sampled genome datasets.

Importantly, it will be essential to understand whether the prophage genes with unknown functions are directly contributing to (or strongly influencing) pneumococcal pathogenesis and virulence. Pneumococcal pathogenesis is complicated and whether or not there is a significant prophage contribution is not yet well understood, but the vast array of prophage DNA within these pneumococcal genomes warrants a detailed exploration.

## Methods

### Compilation of the pneumococcal genome dataset

The study dataset consisted of 482 historical and modern pneumococcal genomes (Table S1) described in our previously published studies^16^,^17^,^25^,^26^,^27^,^28^, but 42 of these were re-sequenced to obtain genome assemblies with fewer contigs than the original assemblies generated some years ago. Full-length prophage sequence detection was difficult if the genomes contained many assembly gaps. In addition, 46 historical pneumococci were added from an isolate collection donated by the Statens Serum Institut, and these were also sequenced as described below. Quality control statistics for all 482 pneumococcal genome sequences are detailed in Table S6.

Pneumococci were cultured using standard protocols and DNA was extracted using the Promega Maxwell^®^ 16 Instrument and Buccal Swab LEV DNA purification kits. Extracted DNA samples were sent to the Oxford Genomics Centre, where libraries were made, genome sequencing was performed on the Illumina^®^ platform, and de novo sequence assemblies were generated using Velvet^29^. Genome assembly quality was further improved using SSPACE and GapFiller^30^,^31^. Genes were annotated using Prokka and sequences were visualised using Artemis^32^,^33^. Pneumococcal sequences and metadata were stored in a BIGSdb database^34^. STs were automatically tagged and defined in BIGSdb via links to the PubMLST website^20^ and CCs were defined using Phyloviz^35^. The 66 representative full-length phage sequences were deposited in GenBank (accession numbers in Table S2). All assembled pneumococcal genome sequences with corresponding metadata can be accessed from the PubMLST website^20^.

### Interrogation of pneumococcal genomes for phage sequences

An initial screen of the first 336 genomes in the dataset using PHAST resulted in a prophage DNA hit rate of 86% among the pneumococcal genomes^36^. Subsets of the data were explored further, which led to the identification of ten newly-discovered prophage sequences. It also became clear that while PHAST was generally accurate in detecting prophage sequence, the identification of specific prophages was suboptimal. Therefore, the entire dataset of 482 pneumococcal genomes was screened using a reference prophage database we compiled and an in-house pipeline that facilitated BLAT searches of the pneumococcal genome dataset for evidence of prophage sequence^37^. The prophage reference sequence database consisted of 81 previously-characterised streptococcal species prophage sequences downloaded from GenBank, published prophage sequences obtained from Timothy Mitchell^13^, plus the ten new prophage sequences we identified in the initial screen.

The screening pipeline returned the three best BLAT hits per pneumococcal genome and each of these was manually examined. This resulted in the identification of seven more new prophage sequences, which were added to our reference prophage database and the entire genome dataset was rescreened. The screens also returned hits to >1 full-length prophage sequences in different locations within a single pneumococcal genome, all of which were manually inspected to confirm poly-lysogeny (the presence of different prophage sequences in the same pneumococcal genome). The screens also frequently revealed >10 Kb highly similar hits to reference prophages, and investigation of some of these long sequence matches revealed that PHAST and our bespoke screens were missing many full-length prophages, so the gene annotation files for all 482 genomes were manually investigated for evidence of prophage sequences. Ultimately, new prophages were defined as those with a nucleotide sequence <98% identical to any other known prophage in the prophage reference sequence database or our study dataset. Extensive cross-referencing and BLAST searching of previously-identified prophages to the many new prophages identified by manual inspection confirmed the presence and novelty of the detected prophages.

Altogether, these analyses revealed three categories of prophage sequences. ‘Full-length’ prophage DNA sequences were defined as those that started with an integrase gene, ended with an amidase or lysin gene and were >28 kb in length. ‘Putatively full-length’ prophages were defined as those that started with an integrase and nearly met the full-length criteria; however, there was an assembly gap at the end (usually at or around *pblB*) such that the last few gene sequences were missing, but these could be found elsewhere in the genome assembly. However, since many genomes contained multiple prophage sequences it was often impossible to reconnect with confidence the two or more prophage sequence fragments bioinformatically, so only the longest contiguous part of the prophage sequence was extracted and labelled as IPPX(n). ‘Partial’ prophage sequences contained a series of contiguous phage-associated genes, may or may not have included an integrase or amidase gene, were between 2–25 Kb in length, and were also labelled as IPPX(n).

### Genomic analyses of phage sequences

93 full-length prophages were identified and the individual gene nucleotide sequences were clustered using Roary set at a 90% similarity threshold^38^. The 66 unique full-length prophage genomes were visually confirmed and reverse-complemented as required in order to be in the same orientation using Artemis. Multiple sequence alignment (MSA) was performed in Geneious version 9.1 (Biomatters Ltd) using the ClustalW algorithm with default parameters (Gap open cost = 15, Gap extend cost = 6.66)^39^. The MSA output was used within Geneious to calculate percentage identity matrices and create gene alignment figures. Tree building was performed using the Jukes–Cantor model with default parameters in FastTree^40^. Roary was used to identify all of the genes present within each prophage cluster and then an in-house Python script was developed to identify those genes found in more than one prophage cluster. Figures were edited using Inkscape^41^.

### RNA-sequencing experiments and analyses

Seven 10 ml tubes of brain-heart infusion broth were inoculated with pneumococcal reference strain PMEN3 and incubated at 37 °C + 5% CO_2_. An aliquot of 0.5 ml broth was removed and the absorbance at OD_600_ was measured at each time point from 0–6 h to measure increased bacterial growth. Mitomycin C (Sigma-Aldrich) was added to the broth culture tubes to a final concentration of 2 μg/ml after 3 h of incubation (OD_600_ ≥ 0.5). Just prior to the addition of mitomycin C at 3 h and after 4, 5 and 6 h of incubation, respectively, broth cultures were removed from the incubator for processing. A 0.5 ml aliquot was used to measure the absorbance and the RNA was stabilised in the remaining 9.5 ml of broth culture by the addition of 19 ml of RNAprotect Bacteria Reagent (Qiagen). RNA was immediately extracted from the samples using the Promega Maxwell^®^ 16 Instrument and LEV simplyRNA Cells purification kit, following the manufacturer’s protocol.

RNA extracts were sent to the Oxford Genomics Centre where library preps were made using RNA-Seq Ribozero kits (Illumina, Inc) and sequencing was performed on the MiSeq (Illumina, Inc). The sequenced forward and reverse reads were paired and mapped to reference genomes using Bowtie2 with the highest sensitivity option^42^. Differential gene expression was assessed in Geneious using the DESeq method^43^. Genes with an adjusted p-value < 0.05 were deemed to be differentially expressed. RNAseq data are provided in Table S7. Sequence data have been deposited in the Gene Expression Omnibus repository with accession numbers GSM2360598-GSM2360607^44^.

## Additional Information

**How to cite this article:** Brueggemann, A. B. *et al*. Pneumococcal prophages are diverse, but not without structure or history. *Sci. Rep.* **7**, 42976; doi: 10.1038/srep42976 (2017).

**Publisher's note:** Springer Nature remains neutral with regard to jurisdictional claims in published maps and institutional affiliations.

## Supplementary Material

Supplementary Figure S1

Supplementary Table S1

Supplementary Table S2

Supplementary Table S3

Supplementary Table S4

Supplementary Table S5

Supplementary Table S6

Supplementary Table S7

## Figures and Tables

**Table 1:** Pneumococcal genes flanking either side of the 93 integrated full-length prophage sequences.

Cat^a^	N^b^	Genes upstream of phage integrase			PI^c^	Genes downstream of phage amidase		
a	35^d^	P-loop-containing kinase	transporter	putative sporulation transcription regulator whiA	I	pyridine nucleotide-disulfide oxidoreductase	ABC transporter permease	lipoprotein
**b**	**7**	transposase	recombination regulator RecX	adenylosuccinate synthetase	**II**	hypothetical protein	cytidine/deoxycytidylate deaminase	deoxyuridine 5’-triphosphate nucleotidohydrolase
**b**	**6**	transposase	recombination regulator RecX	adenylosuccinate synthetase	**II**	DNA-binding protein	hypothetical protein	cytidine/deoxycytidylate deaminase
**b**	**5**	IS1167 transposase	recombination regulator RecX	adenylosuccinate synthetase	**II**	DNA-binding protein	cytidine/deoxycytidylate deaminase	deoxyuridine 5’-triphosphate nucleotidohydrolase
**b**	**3**	IS1167 transposase	recombination regulator RecX	adenylosuccinate synthetase	**II**	DNA-binding protein	hypothetical protein	cytidine/deoxycytidylate deaminase
**b**	**2**	tRNA-Asn	recombination regulator RecX	adenylosuccinate synthetase	**II**	DNA-binding protein	cytidine/deoxycytidylate deaminase	deoxyuridine 5’-triphosphate nucleotidohydrolase
**b**	**2**	tRNA-Asn	recombination regulator RecX	adenylosuccinate synthetase	**II**	DNA-binding protein	hypothetical protein	cytidine/deoxycytidylate deaminase
**b**	**2**	transposase	recombination regulator RecX	adenylosuccinate synthetase	**II**	DNA-binding protein	cytidine/deoxycytidylate deaminase	phage integrase
**b**	**1**	assembly gap	recombination regulator RecX	adenylosuccinate synthetase	**II**	DNA-binding protein	hypothetical protein	cytidine/deoxycytidylate deaminase
**b**	**1**	transposase	recombination regulator RecX	adenylosuccinate synthetase	**II**	hypothetical protein	arginine deiminase	arginine deiminase
**b**	**1**	transposase-like protein, IS630	recombination regulator RecX	adenylosuccinate synthetase	**II**	DNA-binding protein	cytidine/deoxycytidylate deaminase	deoxyuridine 5’-triphosphate nucleotidohydrolase
**b**	**1**	transposase-like protein, IS630	recombination regulator RecX	adenylosuccinate synthetase	**II**	DNA-binding protein	hypothetical protein	cytidine/deoxycytidylate deaminase
**b**	**1**	tRNA-Asn	recombination regulator RecX	adenylosuccinate synthetase	**II**	DNA-binding protein	integrase	assembly gap
**b**	**1**	—	—	assembly gap	**II**	DNA-binding protein	cytidine/deoxycytidylate deaminase	deoxyuridine 5’-triphosphate nucleotidohydrolase
**c**	**3**	recombination regulator RecX	adenylosuccinate synthetase	cytidine/deoxycytidylate deaminase	**III**	deoxyuridine 5’-triphosphate nucleotidohydrolase	phosphoglycerate mutase	DNA repair protein RadA
**c**	**1**	phage lytic amidase	DNA-binding protein	cytidine/deoxycytidylate deaminase	**III**	deoxyuridine 5’-triphosphate nucleotidohydrolase	phosphoglycerate mutase	DNA repair protein RadA
**c**	**1**	DNA-binding protein	hypothetical protein	cytidine/deoxycytidylate deaminase	**III**	deoxyuridine 5’-triphosphate nucleotidohydrolase	phosphoglycerate mutase	DNA repair protein RadA
**c**	**1**	recombination regulator RecX	adenylosuccinate synthetase	cytidine/deoxycytidylate deaminase	**V**	deoxyuridine 5’-triphosphate nucleotidohydrolase	phosphoglycerate mutase	DNA repair protein RadA
**d**	**3**	superoxide dismutase	competence protein CglA	competence protein CglB	**IV**	hypothetical protein	competence protein CglC	competence protein CglD
**e**	**3**	leucyl-tRNA synthetase	acetyltransferase	GNAT family acetyltransferase	**II**	DNA-binding protein	Holliday junction DNA helicase RuvB	hypothetical protein ProS

^a^Cat = pneumococcal genome insertion site category. 13 examples of genomes with miscellaneous prophage integration sites, including assembly gaps on either side of the prophage sequence, are not shown here. A more detailed list of the integration sites and flanking genes may be found in Table S5, which includes the flanking genes associated with the 193 putatively full-length prophage sequences.

^b^N = number of prophages.

^c^PI = prophage integrase group, determined by the nucleotide sequence of the integrase.

^d^Two pneumococcal genomes had assembly gaps after the prophage amidase.

**Table 2:** Associations between full-length and putatively full-length prophages and pneumococcal genetic lineages.

	Prophage frequency (years of isolation)^a^																			
Clonal complex	IPP32 (1977–1988)	MM1 (1984–2002)	IPPX302 (1978–1985)	SPN195_1 (1993–2008)	IPPX3 (1978–1984)	IPPX4 (1978–1987)	3059_06_phage (1978–1985)	IPPX300 (1999–2003)	IPP57 (1992–2005)	IPP12 (1948–2002)	IPP29 (1978–2002)	IPPX328 (1978)	IPP34 (1939–1999)	IPP62 (1999–2006)	IPP67 (1957–1962)	SPN_1873 (1999–2000)	IPPX215 (1999–2006)	IPP8 (1989–2000)	IPPX100 (1965–1966)	Total
1094			7		6	6		5												24
41/1605	14											3								17
81		11																		11
156/162				7																7
199							5										1			6
124							1				4									5
180									5											5
2090																3	1			4
3122/5977																			3	3
113													3							3
15															3					3
439																		2		2
Singleton	1																1			2
66														2						2
217										2										2
615										2										2
236/271/320																		1		1
7232														1						1
68/1656	1																			1
Total	16	11	7	7	6	6	6	5	5	4	4	3	3	3	3	3	3	3	3	101

^a^Only the full-length and putatively full-length prophages present ≥3 times in the study dataset were included here. The years of isolation refer to when the host pneumococci were isolated.
