# Supplementary material for: Pneumococcal prophages are diverse, but not without structure or history
Source: Sci Rep. 2017 Feb 20;7:42976. doi: 10.1038/srep42976 (PMC5317160; doi:10.1038/srep42976)
Supplement: Supplementary Figure S1 [file srep42976-s1.pdf]

Supplementary Information:

Pneumococcal prophages are diverse, but not without structure or history

Angela B Brueggemann, Caroline L Harrold, Reza Rezaei Javan, Andries J van Tonder, Angus J McDonnell and Ben A Edwards

Nuffield Department of Medicine, University of Oxford, United Kingdom

Corresponding author: Angela B Brueggemann (angela.brueggemann@ndm.ox.ac.uk)

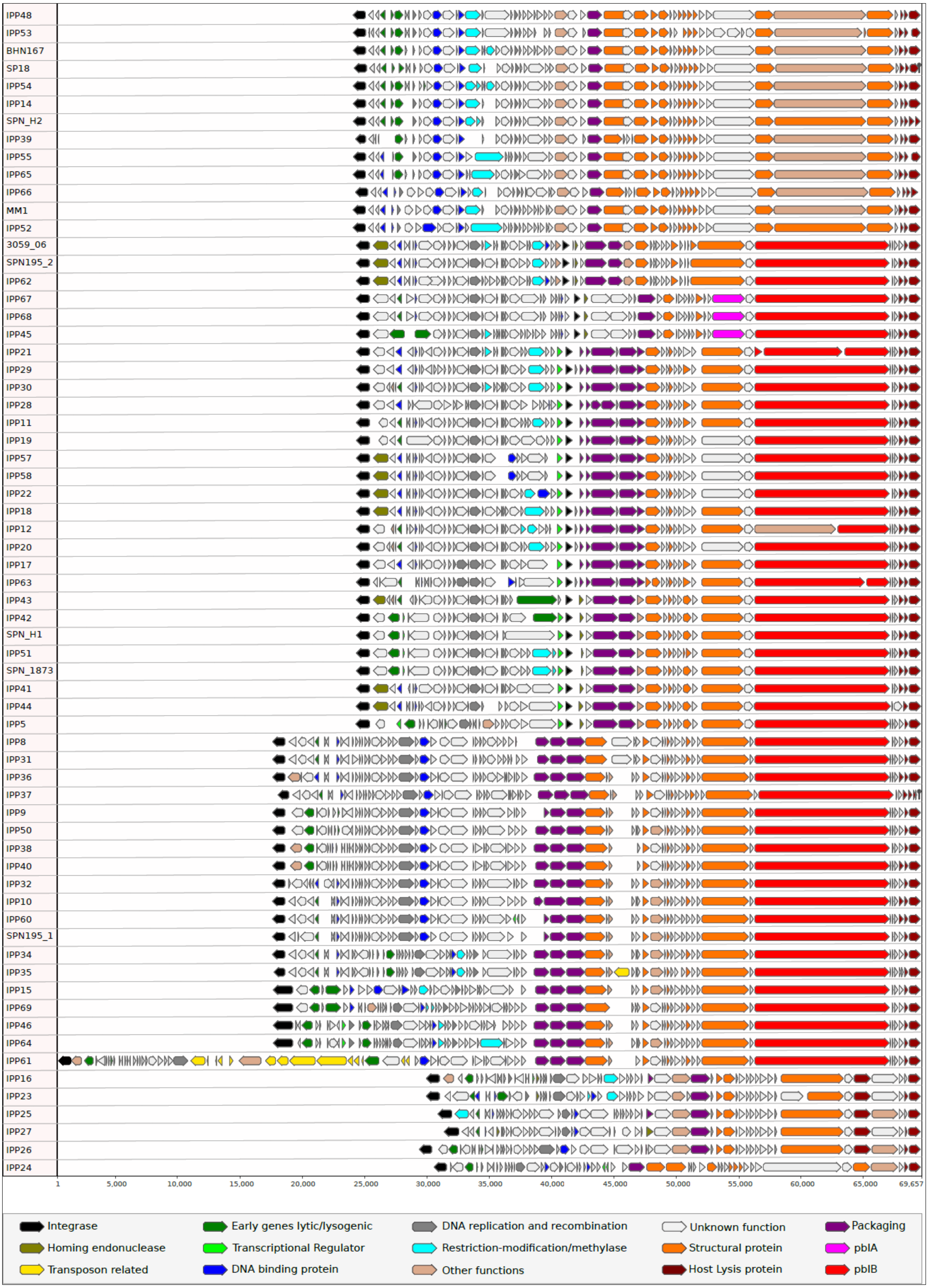

Figure S1. Alignment of 66 representative full-length prophage sequences.
